# Delayed onset of fat embolus in the cerebral venous system after breast and hip augmentation: a case report

**DOI:** 10.1186/s12883-021-02419-x

**Published:** 2021-10-26

**Authors:** Wei Dong, Ding-yuan Wan, Xiang Yang, Min Fu, Xing Liu, Hao Li, Xiao-qi Xie

**Affiliations:** 1 Department of Neuro-intensive Care Unit, West China Hospital, Sichuan University, Chengdu, 610041 China; 2 West China School of Medicine, West China Hospital, Sichuan University, Chengdu, 610041 China; 3 Department of Neurosurgery, West China Hospital, Sichuan University, Chengdu, 610041 China; 4 Department of Intensive Care Unit, Mianyang 404 Hospital, Mianyang, 621000 China

**Keywords:** Cerebral fat embolism, Rare disease, Heparin, Thrombolytic therapy, Case report

## Abstract

**Background:**

Cerebral fat embolism (CFE) is a subtype of fat embolism syndrome which tends to cause ischemic cerebral infarction. Fat embolism in the cerebral venous system have not been reported. We hereby present a rare case of fat embolus formed in the cerebral venous system 10 days after cosmetic surgery, and describe our management of this patient.

**Case presentation:**

A 26-year-old woman with the disturbance of consciousness and recurrent convulsions of the right upper extremity over a 21-h period was admitted to our hospital. The patient was initially diagnosed with haemorrhagic infarction, and cerebral venous thrombosis (CVT) was suspected based on computed tomography (CT). A diagnosis of CFE was confirmed based on surgical findings. Breast and hip augmentation performed 10 days ago was considered the underlying cause. Drug-induced hypothermia, low molecular weight heparin, atorvastatin, dexamethasone, piperacillin/tazobactam, valproic acid, and mannitol were applied. On hospital day 30, she was discharged with a Montreal Cognitive Assessment score of 25.

**Conclusions:**

Fat embolism can occur in the cerebral venous system, and may mimic CVT symptoms rather than CFE symptoms. Early identification of the nature of the embolus is essential. The use of heparin may prevent secondary thrombus formation, and accelerate fat embolus decomposition.

## Background

Cerebral fat embolism (CFE) is a rare manifestation of fat embolism, with an incidence rate of 0.9–2.2% and a mortality rate of up to 10% [1–3]. Long bone fractures are the leading cause of CFE, during which fat from the medullary space is released into the veins [4]. When compared to bone fractures, fat grafting is a much rarer cause that only accounts for ≤0.09% of CFE, but has been causing more cases within the past years due to increase in cosmetic surgery [5–8]. Moreover, this severe adverse side effect has been suspected of being underreported [9].

Typical fat graft procedures include three main stages: fat harvesting, fat processing, and fat injection. Autologous fat is usually harvested through techniques such as vacuum aspiration, syringe aspiration, and surgical excision [10]. The abdomen represents the most common area of choice for fat harvesting, followed by the trochanteric region, and the inner aspects of the thighs and knees [10]. Sedimentation, filtering, washing, and centrifugation are subsequently performed to remove collagen fibres, blood, and debris from the harvested fat [10]. Purified adipocytes are then reinjected into the patient through multiple tunnels in a “fanning-out pattern” [10].

Lipid droplets with diameters < 20 μm can pass through the pulmonary capillaries and embolize in the cerebral arteries over a 24–72-h period. Circulating particles may lead to a classic triad of respiratory disorders, rashes, and changes in mental status [4]. However, the alterations in mental status can vary greatly, ranging from normal consciousness to coma [11–14]. The aforementioned symptoms are predominantly present in patients with CFE in the arterial system, and there are currently no similar reports on CFE in the venous system.

Over the past decades, the diagnosis and treatment of CFE have been recognized as a clinical challenge [11, 13]. Available diagnostic tools for fat embolism syndrome have been tentatively used, but have achieved dissatisfactory results [4, 15, 16]. In a case series of four patients with CFE, none of them satisfied the existing clinical criteria [11]. Current therapies for CFE are mainly symptomatic and supportive [13]. While symptomatic treatment involves heparin and corticosteroids [4], the evidence regarding the use of heparin has been controversial. There is also no consensus on the recommended supportive care for CFE. In a recent review, mild hypothermal therapy and dehydrating agents were recommended [4].

We hereby report a rare case of CFE in the cerebral venous system in a 26-year-old woman, in whom pharmacological hypothermia was applied. Our paper aims to highlight 1) the uncertain mechanisms to which the fat embolus led to cerebral venous thrombosis (CVT) symptoms, and 2) the potential role of heparin in CFE treatment.

## Case presentation

**Fig. 1:**
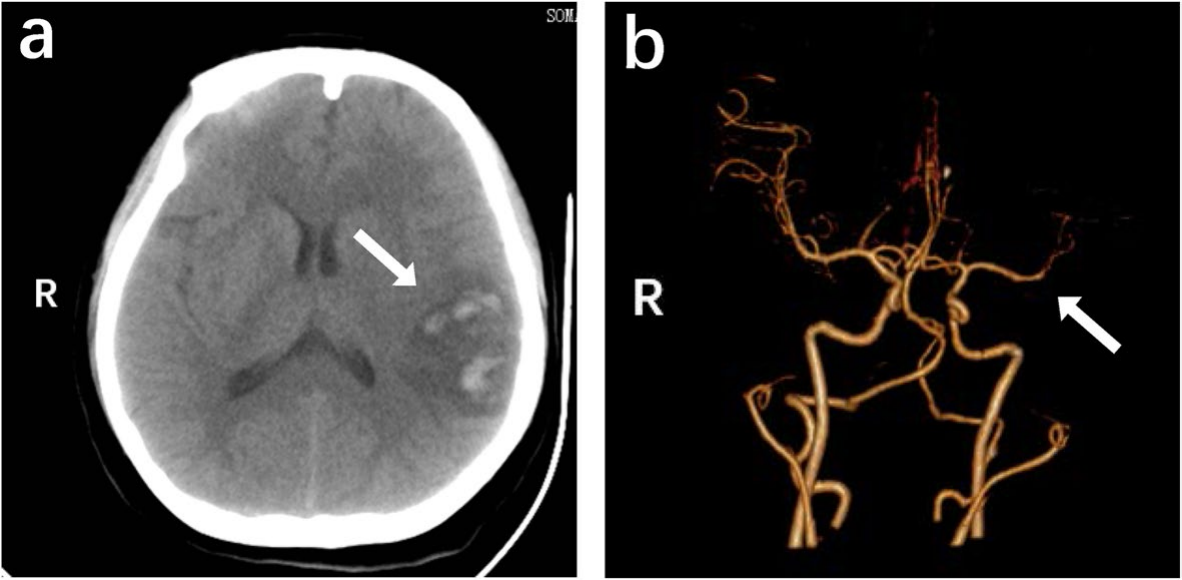
CT/CTA images on admission. **a** Typical haemorrhagic infarction findings detected on CT. **b** The distal left middle cerebral artery appeared vague on CT angiography

**Fig. 2:**
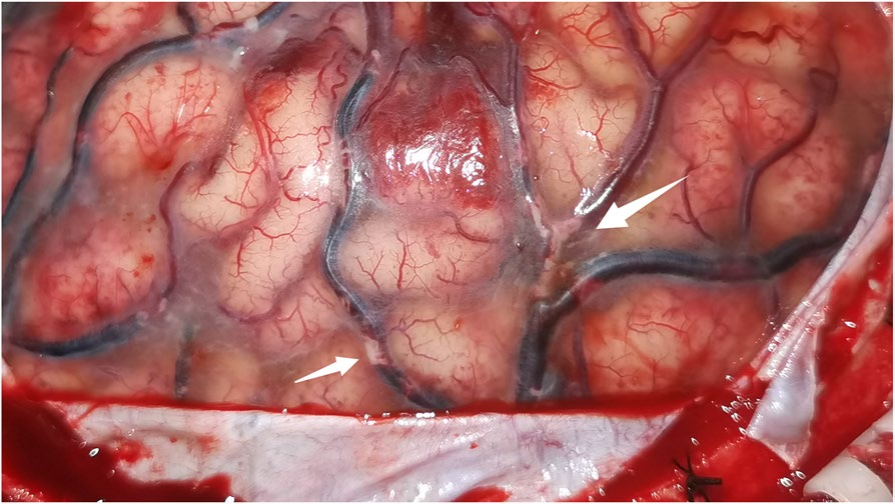
Surgical findings. A large fat embolus was found in the left superficial vein during large trauma craniotomy (HD 2). The dark coloured areas observed on the superficial veins indicated regions of anoxia

A 26-year-old woman with the disturbance of consciousness and recurrent convulsions of the right upper extremity over a 21-h period was admitted to our hospital. The patient interview revealed a history of breast and hip augmentation 10 days ago. On physical examination, she was confused and uncooperative, but showed no signs of rash or dyspnoea. Her Glasgow Coma Scale (GCS) score was E1V1M3, while her vital signs and routine blood test results were normal. No pathological abnormalities were observed on chest computed tomography (CT), transthoracic echocardiography, or transoesophageal echocardiogram. No pulmonary arterial hypertension was demonstrated on lung ultrasonography. A low-density circular structure surrounding an irregular light zone was identified in the left parietal lobe on CT. A haemorrhagic lesion was also detected in the right frontal tip (Fig. 1a). The distal left middle cerebral artery appeared vague on angiography (Fig. 1b). The patient was initially diagnosed with haemorrhagic infarction on hospital day (HD) 1. After 24 h of conservative therapy, her intracranial hypertension worsened, and a distinct midline shift was observed. At this point, her GCS and National Institutes of Health Stroke Scale scores were E1V1M1 and 36, respectively. A large trauma craniotomy was immediately performed, during which a fat embolus was found in the left superficial vein (Fig. 2). The patient was then transferred to a neuro-intensive care unit, and was rediagnosed with cerebral venous embolism according to surgical findings.

Mild hypothermal therapy was initiated on HD 2. The patient was cooled to a target temperature of 35.0 °C using both a hibernate mixture and a cooling blanket. A urinary bladder catheter was used for core body temperature monitoring. A lytic cocktail (100 mg pethidine + 50 mg chlorpromazine + 50 mg promethazine) was administered intravenously after dissolution in 50 mL normal saline. The flow rate was set at 8 mL/h, and was adjusted every hour according to the measured core temperature. The cooling blanket was removed on HD 8, and the lytic cocktail was gradually reduced at a rate of 0.5 °C /h during the rewarming process. On HD 2, after careful evaluation of the haemorrhage risk, subcutaneous low molecular weight heparin (LMWH) was initiated at a daily dose of 6150 AXaIU. Her serum triglyceride levels were well controlled (0.88 mmol/L) during the administration. The drug was discontinued on HD 8, but her cholesterol levels rose to 6.96 mmol/L 3 days later. Atorvastatin was administered on HD 13 to maintain an average serum lipid content. Other symptomatic treatments, including dexamethasone, piperacillin/tazobactam, valproic acid, and mannitol, were administered under regular guidance.

**Fig. 3:**
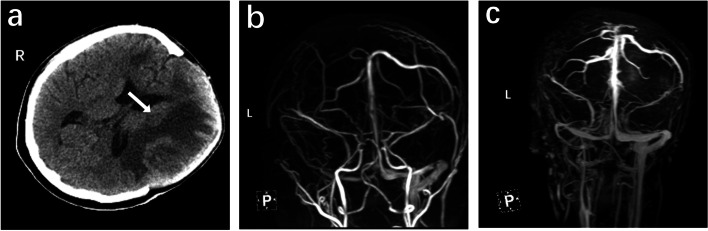
Post-operative brain images. **a** Restoration of brain midline was observed after large craniotomy. Recession of focal necrosis was also demonstrated (HD 4). **b** Stenosis of the superior sagittal, left transverse, and sigmoid sinuses were detected on magnetic resonance venography, suggesting cerebral venous thrombosis (HD 4). **c** Recanalization of the aforementioned sites were shown on HD 20

**Fig. 4:**
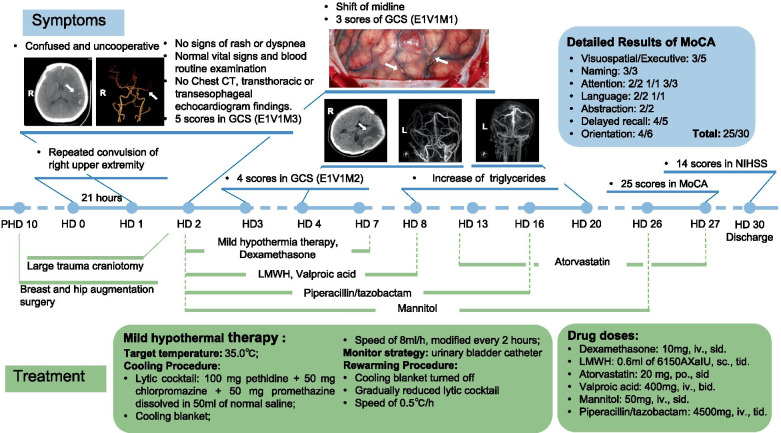
Timeline of the symptoms and management of the patient. PHD, pre-hospital day; HD, hospital day; GCS, Glasgow Coma Scale; NIHSS, National Institutes of Health Stroke Scale; MoCA, Montreal Cognitive Assessment; LMWH, low molecular weight heparin

The midline returned to normal after the surgery, and only slight improvement in neurological function was observed (GCS score: E1V1M2). Brain CT on HD 4 revealed mild recovery of the infarcted area (Fig. 3c). Magnetic resonance venography was also performed, which revealed stenosis of the superior sagittal, left transverse, and sigmoid sinuses (Fig. 3b). Recanalization of the aforementioned vessels and sinuses were observed on magnetic resonance venography on HD 20 (Fig. 3c). Her speech improved on HD 27, which enabled the Montreal Cognitive Assessment to be conducted. A score of 25 was achieved, with two points lost in the visuospatial/executive domain, two points in the orientation domain, and one point in the delayed recall domain. The National Institutes of Health Stroke Scale score was found to be 14, with right sided paralysis greatly contributing to the score. On HD 30, the patient was discharged with a GCS score of 15/15. Details of the symptoms, management, and outcomes of the patient are summarized in Fig. 4.

## Discussion and conclusion

### Cerebral venous embolism

CFE following cosmetic surgery is usually induced by facial fat grafting [6, 17]. Following injection, fats may travel against normal blood flow through the facial artery under continuous pressure. It may then pass through the external carotid artery, and subsequently into the internal carotid artery, resulting in various symptoms [17]. In the setting of other cosmetic surgeries, injected fats tend to be lodged into the pulmonary capillaries, causing pulmonary arterial hypertension. Here, we report a rare case of fat embolism in the cerebral venous system, which led to typical CVT symptoms rather than CFE symptoms. Onset of the disease was 10 days after breast and hip augmentation. Such unusual time gap would not have suggested a causal relationship between the surgery and CFE. However, based on the huge volume of fat embolism observed, an alternative explanation could not be made. Compared to CFE, CVT is a relatively common cause of stroke in young and middle-aged populations [18]. Due to the nature of the embolus, the current case mimicked the symptoms of CVT, but responded well to CFE treatment. CFE is a rare disease that requires further investigation.

### Pathway

A fat embolus was observed in the superficial vein during surgery. Magnetic resonance venography performed on HD 4 revealed stenosis in the superior sagittal, left transverse, and sigmoid sinuses. At these locations, venous drainage was blocked by the embolus, leading to haemorrhagic infarction. Based on the nature of the embolus, we speculated that the fat graft surgery performed 10 days prior was the underlying cause. Nevertheless, we found it challenging to explain not only the formation of the embolus, but also the absence of CFE symptoms such as dyspnoea [4].

The retrograding pathway was considered the most promising mechanism. To examine this possibility, the injection pressure exerted during the cosmetic surgery would be needed. However, there are no published studies on the appropriate pressure needed for fat reinjection in humans. Since hyaluronic acid can serve as a substitute for autologous fat in clinical settings due to their similar physical properties, an estimation based on the relevant data of hyaluronic acid was made [19]. In a recent study, an injection pressure of 684.1 mmHg was needed to push the juvederm through the facial arteries [20]. Moreover, the pressures required for restylane, radiesse and belotero have been reported to be 1081.3 mmHg, 1912.5 mmHg and 617.9 mmHg, respectively [20]. Theoretically, all the aforementioned pressures can be achieved under clinical settings, since they are far lower than the maximum pressure generated by human hands [21]. In addition, these pressures can be achieved with increased flow rate [22]. The pressure needed to inject fat into human tissues has not been studied. However, in an experiment conducted on nude mice, the authors reported that to maintain a speed of 3–5 mL/s, the required pressure can reach 2743.6 mmHg, which is beyond the aforementioned pressures [22]. Under these circumstances, the fat droplets may travel through the inferior vena cava, go against the blood flow of the superior vena cava, and eventually be captured by the cerebral venous system. The lodged fat may not induce clinical symptoms immediately, but can serve as the core for subsequent thrombus formation [8]. The retrograde pathway can thus explain a) the lack of dyspnoea, and b) the delayed onset of other clinical manifestations. It was hence considered the most possible cause of CFE in our case.

We also considered the conventional venous pathway, whereby fat emboli pass through the capillary networks twice — once in the lungs and once in the brain. However, the abundant lipids (100–200 mL) used for fat grafting should have provoked dyspnoea when passing through the pulmonary capillaries, but this was absent during physical examination. Furthermore, no pulmonary arterial hypertension was revealed on lung ultrasonography. In addition, the embolus is less likely to have passed through the cerebral capillaries to subsequently embolize in the cerebral venous system.

The crossed pathway was also discussed and later rejected. Provided this assumption holds, it does not explain how the fat embolus escaped the cerebral capillary sieve. In addition, pathological structures such as patent foramen ovale and signs of left-to-right shunt should have been identified on either transthoracic or transoesophageal echocardiography, yet these were not detected in our case.

The three aforementioned pathways are predominantly based on mechanical theories. Another possible hypothesis conforms to the biochemical theory [4], which proposes that the stress state caused by surgery can lead to endocrine disorders, and subsequently the instability of free fatty acids (FFAs) [23]. The comparatively slower blood flow of the cerebral venous system and circuitous architecture provide a suitable environment for FFA to aggregate into fat droplets and, later, into fat emboli. This may account for the absence of systemic symptoms as they escape the cerebral capillary sieve, and lead to distant symptom onset in the cerebral venous system. An elevation in FFA levels following the cosmetic surgery would have supported this theory, but the relevant information was not obtained.

### Diagnosis

CFE has been recognized as a diagnostic challenge over the past few years [4, 11]. A major reason for such difficulty is the lack of specific manifestations or alterations in laboratory test results [4]. Numerous diagnostic criteria have been proposed, but few have been validated **(**Table 1**)** [4, 24]. Gurd’s criteria, as well as Lindeque’s criteria, focus exclusively on the combination of clinical syndromes [25, 26]. Objective indicators were later added to the modified version of Gurd’s criteria [15]. Schonfeld et al. suggested a quantitative approach based on clinical findings [27], whereby a score of more than five is needed to make a diagnosis [27]. Among these criteria, the modified Gurd’s criteria seems to be the most useful in diagnosing CFE, as it takes into account the neurological alterations and corresponding radiological findings.

**Table 1:** Gurd and Wilson’s criteria, Modified Gurd’s criteria, Lindeque’s criteria and Schonfeld’s criteria for CFE diagnosis

Criteria	Items		Diagnosis
Gurd and Wilson’s [25]	Major • Petechiae • Respiratory symptoms with positive radiographic changes • Cerebral signs unrelated with head injury or any other condition	Minor • Tachycardia • Fever • Retinal changes • Anuria or oliguria • Sudden drop in hemoglobin level • Thrombocytopenia • Fat globule in urine or sputum • High EST	2 major or 1 major + 4 minor
Modified Gurd’s [15]	Major • Petechiae on conjunctiva and upper trunk • PaO_2_<60 at FIO_2_ with or without pulmonary infiltrate on chest X-ray • Altered mentality with multiple cerebral white matter lesion on brain MRI	Minor • HR>100/min • Temperature>38 °C • Platelet<100 × 10^3^/μL • Anemia with coagulopathy or DIC without definite ongoing bleeding site • Anuria or oliguria • Retinal infarct	1 major + 3 minor or 2 major + 2 minor
Lindeque’s [26]	• A sustained PaO_2_<60 mmHg • A sustained PaCO_2_>55 mmHg or a PH<7.3 • A sustained respiratory rate>35/min after adequate sedation • Increased work of breathing and tachycardia combined with anxiety		≥1 item
Schonfeld’s [27]	• Petechiae	5	>5 points
	• X-ray infiltrate on chest	4	
	• Hypoxemia	3	
	• Mental confusion	1	
	• Tachycardia	1	
	• Fever	1	
	• Tachypnea	1	

*Abbreviations*: *EST* erythrocyte sedimentation rate, *PaO2* partial pressure of oxygen, *HR* heart rate, *DIC* disseminated intravascular coagulation, *PaCO2* partial pressure of carbon dioxide

In clinical practice, CFE diagnosis is mainly based on radiological examination. Due to the poor ability of CT in distinguishing soft tissue, it is seldom used to diagnose CFE [8, 11]. Lung CT can reveal non-specific abnormalities of pulmonary arterial hypertension under certain circumstances. Brain CT may show mild oedema and haemorrhage, but these are not indicative of CFE [8]. Magnetic resonance imaging (MRI) represents the most useful diagnostic tool for CFE. In the acute stage, a starfield pattern can be identified on T2-weighted images [4]. Certain MRI sequences such as diffusion-weighted imaging and susceptibility-weighted imaging are more sensitive in detecting such pathological alterations [28]. However, these changes may be found in all types of embolic events. At the subacute stage, both confluent and vasogenic oedemas can be detected [4].

To summarize, there are currently no diagnostic criteria with satisfying sensitivities or specificities. The lack of specific symptoms renders it difficult to diagnose CFE. MRI can merely reveal non-specific findings, and brain CT can only reveal haemorrhagic infarction, which is also non-specific. While CFE was initially suspected, typical signs such as rash or dyspnoea were absent in our patient. The negative lung ultrasonography findings further contributed to the inaccurate initial diagnosis. It was surgical findings that eventually led to our diagnosis of CFE.

### Treatment for CFE

CFE is often self-limiting, but can also be fatal if left untreated [4]. Similar to diagnosis, there is no consensus on the treatment of CFE as well [4, 12, 13, 24]. Current CFE treatments are mainly symptomatic, rather than aetiological. The most common drugs used for CFE include corticosteroids, followed by heparin. It is well known that corticosteroids can significantly reduce the activity of inflammation, thereby blocking the release of various cytokines, including interleukin-1 and -6 [4, 8]. In a meta-analysis, the pooled results indicated that corticosteroid use reduced the risk of infection, but did not influence the mortality rate [29]. Heparin has been proposed as a treatment for CFE, although evidence regarding its use has been controversial [4]. Hepatic lipase, which is released from the vascular bed following heparin injection, may mediate the hydrolysis of triglycerides and the decomposition of fat emboli [30]. Previous research has also suggested that exogenous heparin induces lipoprotein lipase (LPL) release [31]. Circulating LPL promotes FFA release from lipoproteins, which can be removed from the circulation by hepatic uptake [31]. Other rarely used symptomatic treatments include 20% sodium dehydrocholate or low molecular dextran, albumin, soluble saponin, and 5% alcohol glucose solution [4, 24]. The utility of these drugs require further validation. Mechanical thrombectomy is also a possible treatment strategy for CFE [7, 32]. Based on a recent case report, satisfying outcomes were demonstrated in a patient who underwent surgery [32]. Supportive therapy for CFE varies depending on the symptoms presented. These can include mechanical ventilation to maintain adequate oxygen input, dehydrating agents to control intracranial pressure, hypothermal therapy to preserve brain function, and anti-epileptic treatment to prevent seizures [4].

Dexamethasone, LMWH, mild hypothermal therapy, mannitol, valproic acid, atorvastatin, and piperacillin/tazobactam were employed in our case (Fig. 4). Dexamethasone, mild hypothermal therapy, mannitol, and valproic acid were administered based on the previously mentioned reasons. Apart from the aforementioned benefits, LMWH was also used based on the presumption that the fat embolus may provoke further embolus formation and disseminated intravascular coagulation (DIC). A newly published guideline stated that minor intracranial haemorrhages are not an absolute contraindication to heparin [33]. Therefore, after careful evaluation of the haemorrhage risk, heparin was applied. On HD 8, ecchymosis was found on the medial aspect of her thighs. LMWH was hence discontinued. Her triglyceride levels started to increase at HD 8, while her cholesterol level rose to 6.96 mmol/L on HD 11. Atorvastatin was therefore administered on HD 13. Tazoxin was also prescribed to prevent potential infection.

### Limitations

The management of this case had several limitations. From an ex post perspective, MRI should have been performed during the acute phase [12]. This was not performed for two reasons. First, MRI is not as convenient as CT in an emergency setting, during which relieving intracranial hypertension by surgery was the primary concern in our case. Second, MRI is not considered the first imaging choice for detecting haemorrhages in clinical settings [34]. Therefore, it was considered a secondary priority in our situation. In addition, there was no well-defined, approved protocol for the mild hypothermal therapy applied in this case. Moreover, more aggressive therapies such as mechanical thrombectomy could have been employed [32], but was rejected in favour of conservative therapy due to the economic hardship of the family.

## Conclusions

This rare case of cerebral venous fat embolism adds to the current understanding of CFE and CVT. To the best of our knowledge, this is the first report of fat embolism in the cerebral venous system. While the retrograde pathway was proposed as the underlying cause, further explorations are warranted. Dexamethasone, LMWH, mild hypothermal therapy, mannitol, valproic acid, atorvastatin, and piperacillin/tazobactam were administered, and resulted in a positive outcome. Heparin was tentatively used in our case, and subsequent lysis of the fat embolus was confirmed on magnetic resonance venography. We therefore speculate that heparin may prevent secondary thrombus formation as well as DIC, and accelerate fat embolus decomposition.

## Data Availability

All data are available from the corresponding authors upon reasonable requests.

## Abbreviations

CT: Computed tomography
MRI: Magnetic resonance imaging
CVT: Cerebral venous thrombosis
CFE: Cerebral fat embolism
HD: Hospital day
FFA: Free fatty acid
LPL: Lipoprotein lipase
DIC: Disseminated intravascular coagulation
LMWH: Low molecular weight heparin
